# Patterns of Perceived Harms and Benefits of the COVID-19 Outbreak in Hong Kong Adults: A Latent Profile Analysis

**DOI:** 10.3390/ijerph19074352

**Published:** 2022-04-05

**Authors:** Bo-Wen Chen, Wei-Jie Gong, Agnes Yuen-Kwan Lai, Shirley Man-Man Sit, Sai-Yin Ho, Man-Ping Wang, Nancy Xiaonan Yu, Tai-Hing Lam

**Affiliations:** 1 Department of Social and Behavioural Sciences, City University of Hong Kong, Hong Kong, China; sylvia.chan@my.cityu.edu.hk; 2 School of Public Health, The University of Hong Kong, Hong Kong, China; gweijie@connect.hku.hk (W.-J.G.); shirlsit@hku.hk (S.M.-M.S.); syho@hku.hk (S.-Y.H.); hrmrlth@hku.hk (T.-H.L.); 3 School of Nursing, The University of Hong Kong, Hong Kong, China; agneslai@hku.hk

**Keywords:** COVID-19, meaning making, perceived harm, perceived benefit, latent profile analysis

## Abstract

The COVID-19 pandemic caused different types of harms and benefits, but the combined patterns of perceived harms and benefits are unclear. We aimed to identify the patterns of perceived harms and benefits of the COVID-19 outbreak and to examine their associations with socio-demographic characteristics, happiness, and changes in smoking and drinking. A population-based cross-sectional online survey was conducted in May 2020 on Hong Kong adults (N = 4520). Patterns of perceived harms and benefits of COVID-19 were identified using latent profile analysis. Their associations with socio-demographic characteristics, happiness, and changes in smoking and drinking were examined using multinomial logistic regression. We identified three distinct patterns: indifferent (66.37%), harm (13.28%), and benefit (20.35%). Compared with the indifferent subgroup, the harm subgroup was younger, less happy, and had increased drinking, and hence might be at higher risk, whereas the benefit subgroup was more likely to be female, live with one or more cohabitants, have postsecondary education, be happier, and have decreased drinking, and could be more adaptive. Future studies can target the harm subgroup to facilitate their positive adjustments.

## 1. Introduction

Amidst the COVID-19 pandemic, various control measures were implemented by different countries and regions [1]. Hong Kong, the most developed city in China, controlled the first and second waves of the pandemic by public health measures such as mask wearing, hand hygiene, social distancing, school closures, and quarantine from 23 January to 31 May 2020 (around the end of Wave 2) [2,3]. As COVID-19 and control measures gradually presented unprecedented, remarkable economic and health problems, meaning making, a coping strategy to help individuals make sense of a stressor [4], can work as a mechanism of cognitive reappraisal to shape individuals’ perceptions of the COVID-19 outbreak. Therefore, assessing negative and positive perceptions toward the pandemic and associated factors can help us understand whether this is a crisis with detrimental impact or an opportunity for positive changes, which is important for outbreak control and health promotion.

A stressor may be perceived as harmful or beneficial, or both. However, the single focus on either harm or benefit prevails, as these two variables seem to be inversely related. Few studies have examined perceived harms and benefits together in the same individuals. Our survey, “Family amidst COVID-19” (FamCov-1) in May 2020 showed socio-demographic disparities in perceived harms and benefits of COVID-19 in Hong Kong adults [5]. Other studies showed that perceptions toward the stressor were associated with mental health and health-related behavior changes: individuals with higher scores in perceived harms were more likely to report psychological distress and increased unhealthy behaviors (e.g., smoking and drinking), while those with higher scores in perceived benefits reported better well-being and decreased unhealthy behaviors before and amidst COVID-19 [6,7,8].

However, the integration of two theoretical frameworks suggests that individuals’ negative and positive evaluations toward an object can coexist with independence. First, the meaning-making theory assumes that individuals hold global meaning that provides a general orientation system with which to interpret their life experiences. When stressful life events challenge the global meaning, the processes of meaning making initiates for individuals to reappraise and assign situational meaning to them, which reduces the discrepancy between global and situational meaning [4]. In the processes of meaning making, cognitive reappraisal may lead to negative and positive reframing, also known as perceived harms and benefits. Second, although these two variables of perceived harms and benefits seem to represent mutually exclusive extremes of a reframing valence, the dual attitude model suggests the independence of individuals’ negative and positive evaluations of objects [9]. Thus, these reframing ideas can only outweigh, not replace, one another, generating four possible cognitive reappraisal patterns: subgroups with perceptions of low levels of harm and benefit (indifferent), high levels of harm and low levels of benefit (harm), low levels of harm and high levels of benefit (benefit), and high levels of harm and benefit (ambivalent).

Accordingly, studies on opinion formation support these theories by differentiating four subgroups of perceived harms and benefits regarding social events (e.g., nuclear waste repositories [10]) and medication adherence (e.g., diabetes medication adherence [11]). During the 2003 SARS outbreak in Hong Kong, a study focusing on perceived harms and benefits (e.g., personal gain and loss, lifestyle changes, financial situations, and social engagement) reported three patterns: cost (harm, 21.3%), benefit (23.9%), and mixed (ambivalent, 54.8%) [12]. However, it is unclear how patterns of perceived harms and benefits would present amidst the COVID-19 pandemic. We used the keywords of “COVID-19”, “SARS-CoV 2”, “2019 nCoV”, “cognitive patterns”, “reappraisal patterns”, “perceived benefit”, “positive cognition”, “perceived harm”, “perceived cost”, and “negative cognition” to search PubMed and Web of Science up to 25 March 2022 and found no articles on the combined patterns of perceived harms and benefits of COVID-19. The investigation of the possible different patterns has strong implications for developing tailor-made intervention programs to address the needs of each subgroup during the COVID-19 pandemic.

In this quantitative study with a cross-sectional survey among Hong Kong adults, we aimed to identify the patterns of perceived harms and benefits of the COVID-19 outbreak and to examine their associations with socio-demographic characteristics, happiness, and health-related behavior changes (i.e., changes in smoking and drinking). We hypothesized that (a) the study sample would be categorized into four subgroups (i.e., indifferent, harm, benefit, and ambivalent); and (b) the four subgroups would show significant differences in socio-demographic characteristics, happiness, and changes in smoking and drinking.

## 2. Materials and Methods

### 2.1. Study Design and Participants

The present study was based on the data collected from the survey, “Family amidst COVID-19” (FamCov-1), under the Jockey Club Smart Family-Link Project. Details of the survey were reported elsewhere [5]. Briefly, Hong Kong residents aged 18 years or above were invited to participate in a cross-sectional online survey during 26–31 May 2020, when the second wave of the COVID-19 outbreak was under control in Hong Kong. In total, 70,984 invitation emails were distributed by the Hong Kong Public Opinion Research Institute, a well-known survey agency, to its probability—and non-probability-based—panels. The probability-based panel included people randomly selected through telephone surveys and representative of the Hong Kong population, whereas the non-probability-based panel included those who volunteered to join through online registration [13]. Invitations to 20,103 emails were opened, and 4944 participants responded. Because our target population was Hong Kong adults having at least one family member, we excluded those having no family members (*n* = 30) and having more than 30% missing values (*n* = 23), leaving 4891 participants who provided useable data. Ethics approval was granted by the Institutional Review Board of the University of Hong Kong/Hospital Authority Hong Kong West Cluster (Reference number: UW 20-238).

### 2.2. Measurements

#### 2.2.1. Perceived Harms and Benefits of the COVID-19 Outbreak

We designed two questions on perceived harms and benefits as, “What harms or benefits have the COVID-19 outbreak brought you?”, with a structured list of putative perceived harms and benefits of COVID-19 based on literature review and team discussion including 17 and 13 response items, respectively [5,12]. These items were classified in the present analysis under five domains: (a) income (e.g., decreased or increased personal income), work, and study (e.g., decreased or increased efficiency in work or study at home), (b) physical health and prevention (e.g., worse or improved overall physical health and personal hygiene), (c) emotions and coping (e.g., increased or decreased negative emotions and ability to cope with adversities), (d) life changes (e.g., decreased social activity and increased time spent with family), and (e) others. Three additional items included, “no harms”, “no benefits”, and “don’t know/refuse to answer”. From participants’ answers of yes or no (1/0) on each item, we summed the “yes” answers to derive the total scores for perceived harms (17 items, score range 0–17) and benefits (13 items, score range 0–13). We pilot-tested our questions and found pilot participants had no difficulties in answering, suggesting face validity.

#### 2.2.2. Happiness and Changes in Smoking and Drinking

Happiness was assessed using the single item, “How happy do you think you are?”, on an 11-point scale ranging from 0 (very unhappy) to 5 (half and half) to 10 (very happy), which was shown to be reliable and valid in previous studies [14,15]. Changes in smoking and drinking since the outbreak were assessed using two separate questions: “Have your smoking/drinking habits changed since the COVID-19 outbreak?”, with the responses categorized as “non-smokers/non-drinkers”, “decrease (decreased by some or a lot)”, “no change”, and “increase (increased by some or a lot)”.

#### 2.2.3. Socio-Demographic Characteristics

Information on sex, age, number of cohabitants, education, and monthly household income was collected. As in our previous paper [5], age, the number of cohabitants, and education were recoded, household income per person was calculated based on household size and dichotomized into “lower” and “higher” by referring to the size-specific median monthly household income in Hong Kong’s census statistics [16].

### 2.3. Statistical Analysis

We used a latent profile analysis (LPA) based on the total scores of perceived harms and benefits to identify cognitive patterns. LPA is a person-centered approach to classify individuals into different subgroups based on their similar characteristics. We used the following model fit indices to select the best model [17]: (a) lower Akaike information criterion (AIC), Bayesian information criterion (BIC), and sample-adjusted BIC (aBIC); (b) entropy higher than 0.80; (c) statistically significant Lo–Mendell–Rubin likelihood ratio tests (LMR) and bootstrap likelihood ratio tests (BLRT); and (d) a minimal observed subgroup proportion of 5.00% or more. To decide on the optimal number of profiles, we also considered the theoretical interpretation of the four patterns suggested by the dual model.

After determining the number of profiles, we assigned participants to their most likely profiles based on their highest posterior membership probability [17]. Unweighted descriptive statistics were computed and differences across profiles were examined using Chi-square tests or ANOVA.

With the indifferent subgroup as reference, multinomial logistic regression models were used to calculate adjusted odds ratios (ORs) to examine the associations of socio-demographic characteristics, happiness, and changes in smoking and drinking with the identified subgroups. First, the associations between socio-demographic characteristics and subgroup membership were examined with mutual adjustments. Then, the associations of happiness and changes in smoking and drinking with subgroup membership were examined by adjusting for confounding socio-demographic characteristics. A two-sided *p* value of less than 0.05 was considered statistically significant. All statistical analyses were performed using Stata/SE 15.1.

## 3. Results

Because the perceived harms and benefits of the COVID-19 outbreak were essential to the present analysis, 371 participants who reported “don’t know/refused to answer” were excluded. Those who replied “no harms” or “no benefits” were included and coded as 0 in the total scores of perceived harms and benefits. Thus, 4520 participants were included in the final analyses.

Table 1 shows that 56.42% of participants were female, 95.82% aged 18–64 years, 94.38% had one or more cohabitants, 86.59% received postsecondary education, and 70.34% had high monthly household income per person. The mean score of happiness was 5.95 ± 2.12. The majority of participants were nonsmokers (92.72%), while only 41.30% were nondrinkers.

### 3.1. LPA model Identification

**Figure 1:**
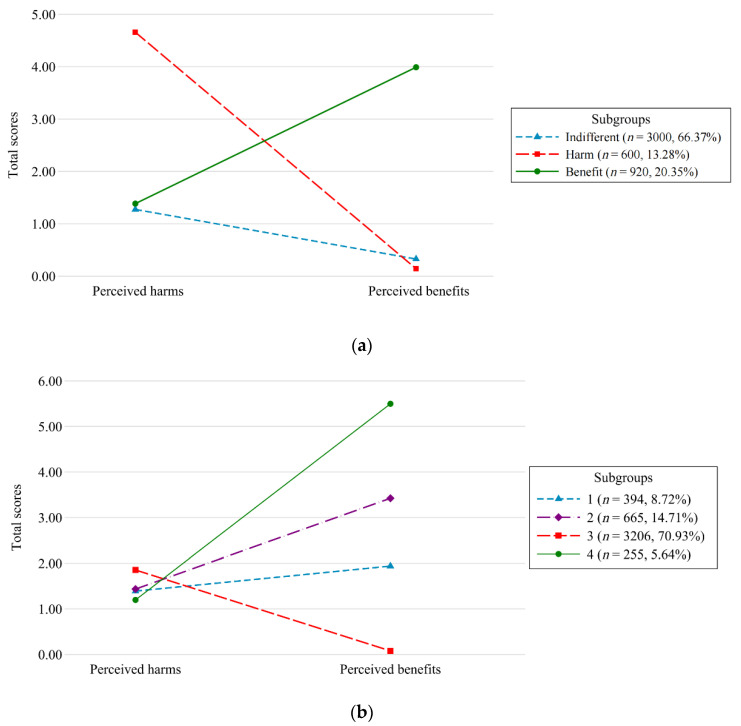
(**a**) The patterns of perceived harms and benefits of the COVID-19 outbreak in three subgroups; (**b**) The patterns of perceived harms and benefits of the COVID-19 outbreak in four subgroups. Note: The *y*-axis indicates the total scores of perceived harms or benefits.

The results of our statistical fit indices for LPA models recommend the three- and four-profile solutions (Table 2). The three-profile model supported our theoretical framework, although the ambivalent subgroup did not appear (Figure 1a). The four-profile solution (a) included a subgroup with a small proportion of 5.64%, leading to a relatively unequal subgroup assignment, and (b) presented consistently low scores on perceived harms but different levels of perceived benefits across all the four subgroups (Figure 1b), showing inconsistency with the independence of perceived harms and benefits suggested by our theoretical framework. Therefore, we rejected the four-profile solution and selected the three-profile solution for further analyses.

Table 3 shows the total scores and the percentage of each item of perceived harms and benefits covering five domains in the total sample and each subgroup. The first subgroup consisted of the largest number of participants (*n* = 3000, 66.37%) and was labeled as the indifferent subgroup because participants reported low scores in both harms (mean = 1.27) and benefits (mean = 0.33). Compared with the indifferent subgroup, the second profile, labeled as the harm subgroup (*n* = 600, 13.28%), reported high scores in harms (mean = 4.66, particularly in the domains of income, work, study, and emotions and coping) but very low scores in benefits (mean = 0.14). The third profile, labeled as the benefit subgroup (*n* = 920, 20.35%), reported low scores in harms (mean = 1.39) but high scores in benefits (mean = 3.99, mainly in the domains of physical health and prevention and emotions and coping). A detailed description of the three subgroups’ characteristics can be found in Table 4. The results of subgroup comparisons using Chi-square tests or ANOVA shows that the three identified subgroups differed significantly in sex, age, number of cohabitants, education, monthly household income per person, happiness, and changes in drinking since the COVID-19 outbreak.

### 3.2. Predictors of the Identified Latent Profiles

Table 5 and Table 6 present the multinomial logistic regression results with the indifferent subgroup as the reference. All ORs for socio-demographic characteristics were mutually adjusted. Since monthly household income per person did not show significant differences in subgroup membership, only sex, age, number of cohabitants, and education were considered potential confounders. In adjusted models, the harm subgroup was younger (less likely to be 65 years or older, OR = 0.41, 95% CI: 0.20–0.85, *p* = 0.017), less happy (less likely to have higher happiness scores, OR = 0.75 per score, 95% CI: 0.72–0.78, *p* < 0.001), and had increased drinking (OR = 1.82, 95% CI: 1.32–2.51, *p* < 0.001). The benefit subgroup was more likely to be female (OR = 1.39, 95% CI: 1.18–1.64, *p* < 0.001), have one or more cohabitants (1–3 cohabitants: OR = 1.69, 95% CI: 1.15–2.48, *p* = 0.008; ≥4: OR = 1.59, 95% CI: 1.04–2.43, *p* = 0.032), postsecondary education (OR = 1.68, 95% CI: 1.27–2.21, *p* < 0.001), higher happiness scores (OR = 1.12 per score, 95% CI: 1.08–1.17, *p* < 0.001), and decreased drinking (OR = 1.34, 95% CI: 1.04–1.74, *p* = 0.025).

## 4. Discussion

We first identified three distinct patterns in perceived harms and benefits (indifferent, harm, and benefit) of COVID-19 in this large sample of adults in the general Hong Kong population. Compared with the indifferent subgroup, we found that the harm subgroup was younger, less happy, and had increased drinking. The benefit subgroup was more likely to be female, live with one or more cohabitants, have postsecondary education, be happier, and have decreased drinking. 

Previous COVID-19 studies conducted in Hong Kong only focused on relevant concepts such as risk perception, preventive behaviors, and vaccination hesitancy [18,19], rather than perceived harms and benefits as we did. A previous qualitative study during the SARS pandemic in Hong Kong identified three subgroups of perceived harms and benefits, including the cost (harm), benefit, and mixed (ambivalent) subgroups [12]. Different from these findings, the ambivalent subgroup with high levels of both perceived harms and benefits did not emerge from our data. There were three possible explanations. First, the coexistence of harm and benefit perceptions may produce an unstable ambivalence with an asymmetric proportion of negative and positive interpretations at a specific time point in the dynamic meaning-making process [20]. Thus, individuals with such unstable ambivalence might vacillate between the negative and positive reframed explanations and present one-side-dominant cognitive perception when they answered our cross-sectional survey. Second, the Hong Kong population experienced such a pandemic for the first time during SARS and responded with mixed perceptions [21] and may obtain psychological preparation for the COVID-19 pandemic with fewer ambivalent evaluations. Third, the traditional Zhongyong (“中庸”) thinking of Confucian doctrine claims to hold a moderate perspective rather than contradictory views toward an object to achieve harmony [22]. To conform with their Zhongyong philosophy and to reduce irrationally contradictory perceptions, our participants might have refrained from reporting simultaneously great numbers of both harms and benefits. Future studies are warranted to test whether this subgroup of ambivalence is also absent in other samples or cultures.

In a cross-sectional survey on a Polish sample, three subgroups were identified using cluster analysis based on three core attitudinal dimensions—affect, cognition, and behaviors—to examine their attitudes toward COVID-19. Only 24.6% of the participants showed an indifferent attitude toward the pandemic because they showed little fear about health and selective compliance with public health restrictions. In contrast, the other two subgroups were cautious (27.4%) and involved (48.1%) because they were concerned about the health of themselves and loved ones and mostly or fully followed the mitigation guidelines [23]. The Polish findings are consistent with our previous findings from the same database of the present study that individuals with fear of COVID-19 not only perceived both harms and benefits, but also paid increased attention to COVID-19 information and delayed physician visits [24,25]. The present study has further provided detailed insight into the combined cognition patterns and found that the indifferent subgroup (66.37%) constituted the majority of our sample. This subgroup reporting minimal harms and benefits could represent the traditional Taoism (“道家”) philosophy to let things take their own course and govern by doing nothing. This indifference is considered a self-defensive mechanism in Taoism [26] and would reduce the individual’s vulnerability to excessive stress and threats amidst COVID-19. As participants responded to the survey during the easing period after the second wave of the COVID-19 outbreak, future studies should investigate whether this indifferent subgroup remains indifferent during new waves of the COVID-19 outbreak. 

The harm subgroup (13.28%) was younger and less happy than the indifferent subgroup, which was also found in a cross-sectional survey in Wuhan and Shanghai, China, showing that older and happier people tended to show a less negative attitude toward COVID-19 [27]. Our harm subgroup had increased drinking, which was consistent with a nationwide study in all 31 provinces of mainland China showing that people who perceived higher COVID-related risks reported increased drinking [28]. A cross-sectional survey among U.S. adults found that increased drinking during the COVID-19 pandemic mainly resulted from increased stress. Participants who perceived more harms than benefits engaged in an unhealthy meaning-making process, which plays a negative role in coping with stress and may lead to increased drinking [29]. Changes in smoking in our harm subgroup were not significant, probably due to the small percentage of smokers in Hong Kong (only 8.88% in the harm subgroup) resulting from successful tobacco control and smoking cessation programs [30].

The benefit subgroup included 20.35% of participants who showed a positive cognitive pattern amidst the pandemic. Supported by previous studies [31], females tended to have a positive perception towards COVID-19, as they were more aware of the severe health consequences of COVID-19 and showed higher acceptance of the life changes brought by the pandemic and related public health measures. Participants having cohabitants and higher education were more likely to be in the benefit subgroup, suggesting that the possession of social and educational resources could help individuals develop a healthy cognitive pattern in response to the pandemic, which was also found in a Spanish survey [32]. In addition, the benefit subgroup was happier, as psychological resources also contribute to positive cognitive reappraisal, or benefits could lead to happiness [20]. More importantly, decreased drinking in the benefit subgroup might have arisen from improved health consciousness, which is consistent with the emerging trend of reduced drinking in the UK amidst the pandemic [33]. Whether such a decrease would be sustained in the future is uncertain and should be monitored.

Characterized by younger, less happy, and increased drinking, our findings underscore the need to identify and assist the harm subgroup (13.28%). Based on our results, future interventions can target this subgroup with meaning-making strategies to facilitate their positive adjustments. For example, a meaning-making intervention was effective in helping cancer patients alter negative appraisal and build psychological resources in stress coping [34]. 

The present study had several limitations. First, as mentioned in our previous research using the same survey data, the participants in our online survey in a large sample within a short period were younger and had higher education levels than the Hong Kong general population. However, our previous studies based on this dataset used the sex, age, and education distribution of the 2019 Hong Kong general population for weighting and found the results of the unweighted and weighted key variables remained similar [15,24]. Second, a self-reporting bias may arise because we relied on asking people to describe their perceived harms and benefits. Because of the lack of an existing assessment tool with good psychometric properties to measure the cognitive evaluations of COVID-19, our self-developed measure needs more evidence of reliability and validity in further studies. Third, a classification error might occur when using the LPA approach, as each participant was assigned to a specific profile according to their posterior probabilities, but their true membership remained unknown. Fourth, the cross-sectional design limited causal inference as the time sequence among variables was not clear. Finally, our results may not be generalizable to other regions that experience a different severity of the COVID-19 outbreak, control policies, and health and socio-economic contexts.

## 5. Conclusions

By profiling cognitive perceptions of the COVID-19 pandemic among Hong Kong adults, this study identified three distinct patterns of perceived harms and benefits related to the pandemic, including the indifferent, harm, and benefit subgroups. To further characterize each pattern with socio-economic status, happiness, and behavioral changes, our study showed that compared with the indifferent subgroup, the harm subgroup was younger, less happy, and had increased drinking, and hence might be at higher risk, whereas the benefit subgroup was more likely to be female, live with one or more cohabitants, have postsecondary education, be happier, have decreased drinking, and could be more adaptive. Our findings have implications for health promotion during the pandemic by developing specific support strategies after taking account of the distinct characteristics of the subgroups in need. For instance, future studies are recommended to detect the at-risk harm subgroup and provide age-sensitive mental health and behavioral modification programs to facilitate their positive adjustment.

## Figures and Tables

**Table 1:** The characteristics of the study sample, unweighted (N = 4520).

	*n* (%) or Mean ± Standard Deviation
Socio-demographic characteristics	
Sex	
Male	1970 (43.58)
Female	2550 (56.42)
Age (years)	
18–24	203 (4.49)
25–44	2272 (50.27)
45–64	1856 (41.06)
≥65	189 (4.18)
Number of cohabitants	
0 (living alone)	250 (5.62)
1–3	3380 (76.04)
≥4	815 (18.34)
Education	
Secondary or below	603 (13.41)
Postsecondary	3892 (86.59)
Monthly household income per person	
Low (≤HK median)	1175 (29.66)
High (>HK median)	2787 (70.34)
Happiness ^a^	5.95 ± 2.12
Changes in smoking since the COVID-19 outbreak	
Nonsmokers	4167 (92.72)
Decrease	76 (1.69)
No change	182 (4.05)
Increase	69 (1.54)
Changes in drinking since the COVID-19 outbreak	
Nondrinkers	1433 (41.30)
Decrease	288 (8.30)
No change	1440 (41.50)
Increase	309 (8.90)

Notes: Missing data were excluded. ^a^ Happiness: a single item rating from 0 (very unhappy), 5 (half and half), to 10 (very happy).

**Table 2:** Results of statistical fit indices of latent profile analysis models.

Model	AIC	BIC	aBIC	LMR_ *p*	BLRT_ *p*	Entropy	Composition
One profile	34,729.66	34,755.32	34,742.61				
Two profile	32,029.73	32,074.64	32,052.40	<0.01	<0.01	0.94	0.80/0.20
Three profile	31,685.71	31,749.87	31,718.09	<0.01	<0.01	0.79	0.66/0.14/0.20
Four profile	28,571.95	28,655.36	28,614.05	<0.01	<0.01	0.98	0.09/0.15/0.71/0.05
Five profile	29,476.05	29,578.71	29,527.87	1.00	1.00	0.89	0.00/0.13/0.12/0.58/0.17

Notes: Smaller Akaike information criterion (AIC), Bayesian information criterion (BIC), and sample-adjusted Bayesian information criterion (aBIC) indicate better model fitness. Significant *p*-values (*p* < 0.05) of Lo–Mendell–Rubin likelihood ratio tests (LMR) and bootstrap likelihood ratio test (BLRT) indicate the fitness of the *k* profile model improved compared with the (*k* − 1) profile model. Greater entropy values indicate better classification, with an entropy value >0.80 showing a relatively high classification accuracy. The composition indicates each subgroup’s percentage, with a minimal observed group size of 5% or more showing relatively equal group assignment.

**Table 3:** Profile characteristics of participants’ responses on each item of perceived harms and benefits in the total sample and each subgroup, unweighted, *n* (%) or Mean ± Standard deviation (N = 4520).

	Total	Indifferent Subgroup (*n* = 3000)	Harm Subgroup (*n* = 600)	Benefit Subgroup (*n* = 920)	*p*-Value
Perceived harms					
Total scores	1.75 ± 1.63	1.27 ± 1.13	4.66 ± 1.01	1.39 ± 1.38	<0.001
Income, work, and study					
Decreased personal income	1334 (29.51)	759 (25.30)	367 (61.17)	208 (22.61)	<0.001
Decreased efficiency in work/study at home	1021 (22.59)	514 (17.13)	354 (59.00)	153 (16.63)	<0.001
Physical health and prevention					
Worse overall physical health	337 (7.46)	117 (3.90)	191 (31.83)	29 (3.15)	<0.001
Increased common colds	29 (0.64)	5 (0.17)	21 (3.50)	3 (0.33)	<0.001
Worse personal hygiene	22 (0.49)	4 (0.13)	18 (3.00)	0 (0.00)	<0.001
Delayed seeing a doctor	462 (10.22)	167 (5.57)	199 (33.17)	96 (10.43)	<0.001
Worse knowledge of epidemic prevention	6 (0.13)	0 (0.00)	5 (0.83)	1 (0.11)	<0.001
Emotions and coping					
Increased negative emotions	2002 (44.29)	1146 (38.20)	570 (95.00)	286 (31.09)	<0.001
Induced anxiety	1635 (36.17)	759 (25.30)	555 (92.50)	321 (34.89)	<0.001
Induced depression	626 (13.85)	145 (4.83)	376 (62.67)	105 (11.41)	<0.001
Decreased ability to cope with adversities	80 (1.77)	9 (0.30)	68 (11.33)	3 (0.33)	<0.001
Life changes					
Cannot travel	35 (0.77)	18 (0.60)	10 (1.67)	7 (0.76)	0.03
Decreased social activity	55 (1.22)	27 (0.90)	11 (1.83)	17 (1.85)	0.02
Decreased outing	29 (0.64)	15 (0.50)	6 (1.00)	8 (0.87)	0.23
Changed plans	28 (0.62)	14 (0.47)	7 (1.17)	7 (0.76)	0.11
Increased expenditure on epidemic prevention supplies	48 (1.06)	29 (0.97)	10 (1.67)	9 (0.98)	0.30
Others	144 (3.19)	95 (3.17)	27 (4.50)	22 (2.39)	0.07
Perceived benefits					
Total scores	1.05 ± 1.67	0.33 ± 0.68	0.14 ± 0.48	3.99 ± 1.09	<0.001
Income, work, and study					
Increased personal income	52 (1.15)	16 (0.53)	1 (0.17)	35 (3.80)	<0.001
Increased efficiency in work/study at home	376 (8.32)	121 (4.03)	7 (1.17)	248 (26.96)	<0.001
Physical health and prevention					
Improved overall physical health	539 (11.92)	59 (1.97)	4 (0.67)	476 (51.74)	<0.001
Decreased common colds	584 (12.92)	75 (2.50)	5 (0.83)	504 (54.78)	<0.001
Improved personal hygiene	1041 (23.03)	241 (8.03)	20 (3.33)	780 (84.78)	<0.001
Increased knowledge of epidemic prevention	1159 (25.64)	323 (10.77)	30 (5.00)	806 (87.61)	<0.001
Emotions and coping					
Decreased negative emotions	87 (1.92)	7 (0.23)	0 (0.00)	80 (8.70)	<0.001
Increased positive emotions	158 (3.50)	15 (0.50)	0 (0.00)	143 (15.54)	<0.001
Increased ability to cope with adversities	600 (13.27)	76 (2.53)	11 (1.83)	513 (55.76)	<0.001
Life changes					
Increased leisure time	31 (0.69)	15 (0.50)	1 (0.17)	15 (1.63)	<0.001
Increased private time	21 (0.46)	10 (0.33)	2 (0.33)	9 (0.98)	0.04
Increased time spent with family	24 (0.53)	9 (0.30)	2 (0.33)	13 (1.41)	<0.001
Others	60 (1.33)	15 (0.50)	3 (0.50)	42 (4.57)	<0.001

**Table 4:** The characteristics of socio-demographics, happiness, and changes in smoking and drinking across three subgroups, unweighted, *n* (%) or Mean ± Standard deviation (N = 4520).

	Indifferent Subgroup (*n* = 3000)	Harm Subgroup (*n* = 600)	Benefit Subgroup (*n* = 920)	*p*-Value	Effect Size ^a^
Socio-demographic characteristics					
Sex					
Male	1350 (45.00)	278 (46.33)	342 (37.17)	<0.001	0.07
Female	1650 (55.00)	322 (53.67)	578 (62.83)		
Age (years)					
18–24	122 (4.07)	36 (6.00)	45 (4.89)	0.03	0.04
25–44	1, 478 (49.27)	318 (53.00)	476 (51.74)		
45–64	1, 270 (42.33)	230 (38.33)	356 (38.70)		
≥65	130 (4.33)	16 (2.67)	43 (4.67)		
Number of cohabitants					
0 (living alone)	186 (6.31)	30 (5.11)	34 (3.73)	0.04	0.03
1–3	2226 (75.53)	440 (74.96)	714 (78.38)		
≥4	535 (18.15)	117 (19.93)	163 (17.89)		
Education					
Secondary or below	438 (14.67)	85 (14.21)	80 (8.77)	<0.001	0.07
Postsecondary	2547 (85.33)	513 (85.79)	832 (91.23)		
Monthly household income per person					
Low (≤HK median)	794 (30.09)	164 (32.60)	217 (26.46)	0.04	0.04
High (>HK median)	1845 (69.91)	339 (67.40)	603 (73.54)		
Happiness ^b^	6.04 ± 2.06	4.66 ± 2.26	6.49 ± 1.90	<0.001	0.06
Changes in smoking since the COVID-19 outbreak					
Nonsmokers	2764 (92.69)	544 (91.12)	859 (93.88)	0.32	0.03
Decrease	46 (1.54)	14 (2.35)	16 (1.75)		
No change	128 (4.29)	26 (4.36)	28 (3.06)		
Increase	44 (1.48)	13 (2.18)	12 (1.31)		
Changes in drinking since the COVID-19 outbreak					
Nonsmokers	957 (38.97)	188 (37.52)	288 (40.39)	<0.001	0.08
Decrease	296 (12.05)	76 (15.17)	116 (16.27)		
No change	1010 (41.12)	166 (33.13)	264 (37.03)		
Increase	193 (7.86)	71 (14.17)	45 (6.31)		

Notes: Missing data were excluded. ^a^ Effect size: Cramer’s V for categorical variables: small, 0.10–0.30; medium, 0.30–0.50; large, ≥0.50. Partial η^2^ for continuous variables: small, 0.10–0.25; medium, 0.25–0.40; large, ≥0.50. ^b^ Happiness: a single item rating from 0 (very unhappy), 5 (half and half), to 10 (very happy).

**Table 5:** Associations of socio-demographic characteristics with identified subgroups of perceived harms and benefits (N = 4520).

	Adjusted OR (95% CI) ^a^	
	Harm vs. Indifferent (Ref.)	Benefit vs. Indifferent (Ref.)
Sex		
Male	1	1
Female	0.96 (0.79, 1.16)	1.39 (1.18, 1.64) ***
Age (years)		
18–24	1	1
25–44	0.79 (0.50, 1.25)	0.80 (0.53, 1.19)
45–64	0.65 (0.40, 1.03)	0.78 (0.52, 1.17)
≥65	0.41 (0.20, 0.85) *	1.06 (0.62, 1.80)
Number of cohabitants		
0 (living alone)	1	1
1–3	1.32 (0.84, 2.08)	1.69 (1.15, 2.48) **
≥4	1.49 (0.91, 2.43)	1.59 (1.04, 2.43) *
Education		
Secondary or below	1	1
Postsecondary	0.87 (0.66, 1.15)	1.68 (1.27, 2.21) ***
Household monthly income per month		
Low (≤HK median)	1	1
High (>HK median)	0.90 (0.72, 1.11)	1.16 (0.97, 1.40)

Notes: Ref., Reference subgroup. ^a^ Mutually adjusted by each other. * *p* < 0.05, ** *p* < 0.01, and *** *p* < 0.001.

**Table 6:** Associations of happiness and changes in smoking and drinking with identified subgroups of perceived harms and benefits (N = 4520).

	Adjusted OR (95% CI) ^a^	
	Harm vs. Indifferent (Ref.)	Benefit vs. Indifferent (Ref.)
Happiness ^b^	0.75 (0.72, 0.78) ***	1.12 (1.08, 1.17) ***
Changes in smoking since the COVID-19 outbreak		
Nonsmokers	1	1
Decrease	1.32 (0.69, 2.52)	1.29 (0.72, 2.31)
No change	1.00 (0.64, 1.56)	0.81 (0.53, 1.25)
Increase	1.52 (0.81, 2.86)	1.06 (0.55, 2.04)
Changes in drinking since the COVID-19 outbreak		
Nondrinkers	1	1
Decrease	1.23 (0.91, 1.67)	1.34 (1.04, 1.74) *
No change	0.81 (0.64, 1.02)	0.90 (0.75, 1.10)
Increase	1.82 (1.32, 2.51) **	0.79 (0.55, 1.13)

Notes: Ref., Reference subgroup. ^a^ Adjusted by sex, age, number of cohabitants, and education. ^b^ Happiness: a single item rating from 0 (very unhappy), 5 (half and half), to 10 (very happy). * *p* < 0.05, ** *p* < 0.01, and *** *p* < 0.001.

## Data Availability

The data presented in this study are available on request from the corresponding authors. The data are not publicly available because our analyses and paper writing on the results are in progress.

## References

[1] World Health Organization Considerations for Implementing and Adjusting Public Health and Social Measures in the Context of COVID-19. https://www.who.int/publications/i/item/considerations-in-adjusting-public-health-and-social-measures-in-the-context-of-covid-19-interim-guidance.

[2] Cheng K.K., Lam T.H., Leung C.C. (2020). Wearing face masks in the community during the COVID-19 pandemic: Altruism and solidarity. Lancet.

[3] Cowling B.J., Ali S.T., Ng T.W.Y., Tsang T.K., Li J.C.M., Fong M.W., Liao Q., Kwan M.Y.W., Lee S.L., Chiu S.S. (2020). Impact assessment of non-pharmaceutical interventions against coronavirus disease 2019 and influenza in Hong Kong: An observational study. Lancet Public Health.

[4] Park C.L. (2010). Making sense of the meaning literature: An integrative review of meaning making and its effects on adjustment to stressful life events. Psychol. Bull..

[5] Wong B.Y., Lam T.H., Lai A.Y., Wang M.P., Ho S.Y. (2021). Perceived benefits and harms of the COVID-19 pandemic on family well-being and their sociodemographic disparities in Hong Kong: A cross-sectional study. Int. J. Environ. Res. Public Health.

[6] Doron J., Trouillet R., Maneveau A., Ninot G., Neveu D. (2015). Coping profiles, perceived stress and health-related behaviors: A cluster analysis approach. Health Promot. Int..

[7] Yang Z., Ji L.J., Yang Y., Wang Y., Zhu L., Cai H. (2021). Meaning making helps cope with COVID-19: A longitudinal study. Pers. Individ. Dif..

[8] Miao M., Zheng L., Wen J., Jin S., Gan Y. (2021). Coping with coronavirus disease 2019: Relationships between coping strategies, benefit finding and well-being. Stress Health.

[9] Wilson T.D., Lindsey S., Schooler T.Y. (2000). A model of dual attitudes. Psychol. Rev..

[10] Seidl R., Moser C., Stauffacher M., Krütli P. (2013). Perceived risk and benefit of nuclear waste repositories: Four opinion clusters. Risk Anal..

[11] Shiyanbola O.O., Unni E., Huang Y.M., Lanier C. (2018). Using the extended self-regulatory model to characterise diabetes medication adherence: A cross-sectional study. BMJ Open.

[12] Cheng C., Wong W.M., Tsang K.W. (2006). Perception of benefits and costs during SARS outbreak: An 18-month prospective study. J. Consult. Clin. Psychol..

[13] Hong Kong Public Opinion Research Institute HKPOP Panel. https://www.pori.hk/team-members.html?lang=en.

[14] Abdel-Khalek A. (2006). Measuring happiness with a single-item scale. Soc. Behav. Pers..

[15] Gong W.J., Wong B.Y., Ho S.Y., Lai A.Y., Zhao S.Z., Wang M.P., Lam T.H. (2021). Family e-Chat group use was associated with family wellbeing and personal happiness in Hong Kong adults amidst the COVID-19 pandemic. Int. J. Environ. Res. Public Health.

[16] Hong Kong Census and Statistics Department Median Monthly Domestic Household Income of Economically Active Households by Household Size. https://www.censtatd.gov.hk/hkstat/sub/sp150.jsp?productCode=D5250038.

[17] Muthén L., Muthén B. (2010). Mplus User’s Guide.

[18] Luk T.T., Zhao S., Wu Y., Wong J.Y., Wang M.P., Lam T.H. (2021). Prevalence and determinants of SARS-CoV-2 vaccine hesitancy in Hong Kong: A population-based survey. Vaccine.

[19] Xu Y., Chen H.F., Yeung W.K.J., Hsieh C.W., Yuan H.Y., Chang L.Y. (2021). Health-promoting behaviors, risk perceptions, and attention to COVID-19-related information: Comparing people’s responses to the COVID-19 pandemic across times of Chinese New Year and Summer 2020 in Hong Kong. Front. Public Health.

[20] Snyder A.I., Tormala Z.L. (2017). Valence asymmetries in attitude ambivalence. J. Pers. Soc. Psychol..

[21] Cheng S.K.W., Chong G.H.C., Chang S.S.Y., Wong C.W., Wong C.S.Y., Wong M.T.P., Wong K.C. (2006). Adjustment to severe acute respiratory syndrome (SARS): Roles of appraisal and post-traumatic growth. Psychol. Health.

[22] Hou Y., Xiao R., Yang X., Chen Y., Peng F., Zhou S., Zeng X., Zhang X. (2020). Parenting style and emotional distress among Chinese college students: A potential mediating role of the Zhongyong thinking style. Front. Psychol..

[23] Boguszewski R., Makowska M., Podkowińska M. (2021). A typology of Poles’ attitudes toward COVID-19 during the first wave of the pandemic. Int. J. Environ. Res. Public Health.

[24] Sit S.M., Lam T.H., Lai A.Y., Wong B.Y., Wang M.P., Ho S.Y. (2021). Fear of COVID-19 and its associations with perceived personal and family benefits and harms in Hong Kong. Transl. Behav. Med..

[25] Lai A.Y.K., Sit S.M.M., Wu S., Wang M.P., Wong B.Y., Ho S.Y., Lam T.H. (2021). Associations of delay in doctor consultation with COVID-19 related fear, attention to information, and fact-checking. Front. Public Health.

[26] Yip K.S. (2004). Taoism and its impact on mental health of the Chinese communities. Int. J. Soc. Psychiatry.

[27] Qian M., Wu Q., Wu P., Hou Z., Liang Y., Cowling B.J., Yu H. (2020). Anxiety levels, precautionary behaviours and public perceptions during the early phase of the COVID-19 outbreak in China: A population-based cross-sectional survey. BMJ Open.

[28] Yan A.F., Sun X., Zheng J., Mi B., Zuo H., Ruan G., Hussain A., Wang Y., Shi Z. (2020). Perceived risk, behavior changes and health-related outcomes during COVID-19 pandemic: Findings among adults with and without diabetes in China. Diabetes Res. Clin. Pract..

[29] Grossman E.R., Benjamin-Neelon S.E., Sonnenschein S. (2020). Alcohol consumption during the COVID-19 Pandemic: A cross-sectional survey of US adults. Int. J. Environ. Res. Public Health.

[30] Li W.H.C., Ho K.Y., Wang M.P., Cheung D.Y.T., Lam K.K.W., Xia W., Cheung K.Y., Wong C.K.H., Chan S.S.C., Lam T.H. (2020). Effectiveness of a brief self-determination theory-based smoking cessation intervention for smokers at emergency departments in Hong Kong: A randomized clinical trial. JAMA Intern. Med..

[31] Galasso V., Pons V., Profeta P., Becher M., Brouard S., Foucault M. (2020). Gender differences in COVID-19 attitudes and behavior: Panel evidence from eight countries. Proc. Natl. Acad. Sci. USA.

[32] Losada-Baltar A., Jiménez-Gonzalo L., Gallego-Alberto L., Pedroso-Chaparro M.D.S., Fernandes-Pires J., Márquez-González M. (2021). “We are staying at home.” Association of self-perceptions of aging, personal and family resources, and loneliness with psychological distress during the lockdown period of COVID-19. J. Gerontol. B Psychol. Sci. Soc. Sci..

[33] Gibbons B. Beer Drinkers More Health Conscious Since Lockdown with Many Now Choosing Low-Alcohol Options. https://www.walesonline.co.uk/whats-on/food-drink-news/beer-drinkers-more-health-conscious-18824786.

[34] Lee V., Robin Cohen S., Edgar L., Laizner A.M., Gagnon A.J. (2006). Meaning-making intervention during breast or colorectal cancer treatment improves self-esteem, optimism, and self-efficacy. Soc. Sci. Med..

